# Ileal Transposition Surgery Decreases Fat Mass and Improves Glucose Metabolism in Diabetic GK Rats: Possible Involvement of FGF21

**DOI:** 10.3389/fphys.2018.00191

**Published:** 2018-03-09

**Authors:** Kemin Yan, Weijie Chen, Huijuan Zhu, Guole Lin, Hui Pan, Naishi Li, Linjie Wang, Hongbo Yang, Meijuan Liu, Fengying Gong

**Affiliations:** ^1^Key Laboratory of Endocrinology of National Health and Family Planning Commission, Department of Endocrinology, Peking Union Medical College Hospital, Chinese Academy of Medical Science and Peking Union Medical College, Beijing, China; ^2^Department of Surgery, Peking Union Medical College Hospital, Chinese Academy of Medical Science and Peking Union Medical College, Beijing, China

**Keywords:** ileal transposition (IT) surgery, FGF21, white adipose tissue (WAT), glucose metabolism, Goto-Kakizaki (GK) rats

## Abstract

**Objective:** Ileal transposition (IT) surgery has been reported to improve glucose and lipid metabolism, and fibroblast growth factor 21 (FGF21) is a powerful metabolic regulator. In the present study, we aimed to investigate the effects of IT surgery on metabolism and its possible relationship with the FGF21 signaling pathway in diabetic Goto-Kakizaki (GK) rats.

**Methods:** Ten-week-old male GK rats were subjected to IT surgery with translocation of a 10 cm ileal segment to the proximal jejunum (IT group) or sham surgery without the ileum transposition (Sham-IT group). Rats in the no surgery group did not receive any surgical intervention. Six weeks later, body weight, fat mass, fasting blood glucose (FBG), and serum levels of FGF21 and leptin were measured. The expression of the FGF21 signaling pathway and white adipose tissue (WAT) browning-related genes in the WAT and liver were evaluated by real-time reverse transcription polymerase chain reaction (RT-qPCR) and western blot.

**Results:** IT surgery significantly decreased the body weights and FBG levels and increased the insulin sensitivity of GK rats. The total WAT mass of the IT rats showed a 41.5% reduction compared with the Sham-IT rats, and serum levels of FGF21 and leptin of the IT rats decreased by 26.3 and 61.7%, respectively (all *P* < 0.05). The mRNA levels of fibroblast growth factor receptor 1 (FGFR1) and its co-receptor β klotho (KLB) in the perirenal WAT (pWAT) of the IT rats were 1.4- and 2.4-fold that of the Sham-IT rats, respectively, and the FGFR1 protein levels were 1.7-fold of the Sham-IT rats (all *P* < 0.05). In accordance with the pWAT, the protein levels of FGFR1 and KLB in the epididymal WAT (eWAT) of the IT rats notably increased to 3.0- and 3.9-fold of the Sham-IT rats (*P* < 0.05). Furthermore, uncoupling protein 1 (UCP1) protein levels in the eWAT and pWAT of the IT rats also increased to 2.2- and 2.3-fold of the Sham-IT rats (*P* < 0.05). However, the protein levels of FGFR1 and KLB in the subcutaneous WAT (sWAT) of the IT rats decreased by 34.4 and 72.1%, respectively, compared with the Sham-IT rats (*P* < 0.05). In addition, the protein levels of FGF21 and KLB in the livers of IT rats were 3.9- and 2.3-fold of the Sham-IT rats (all *P* < 0.05).

**Conclusions:** IT surgery significantly decreased fat mass and improved glucose metabolism in diabetic GK rats. These beneficial roles of IT surgery were probably associated with its stimulatory action on the expression of FGFR1 and KLB in both the eWAT and the pWAT, thereby promoting UCP1 expression in these tissues.

## Introduction

The prevalence of type 2 diabetes mellitus (T2DM) has increased worldwide. In 2014, the worldwide estimates of diabetes were as high as 422 million (WHO, 2016), and the number of people with the disease has been predicted to be 522 million by 2030 (Whiting et al., 2011). Bariatric surgery, also known as metabolic surgery, has been reported to improve T2DM effectively; therefore, it is recommended as a reasonable approach for T2DM treatment (Dixon et al., 2012; Moncada et al., 2016). There are different types of bariatric surgery procedures, and several new metabolic procedures have been introduced in recent years. Among these procedures, ileal transposition (IT) is a novel surgical technique that involves translocation of a segment of the ileum proximally into the upper jejunum without altering the length of the gastrointestinal tract or gastric restriction (Pok and Lee, 2014). IT surgery has been reported to induce weight loss, improve glucose and lipid metabolism and increase insulin sensitivity in diabetic or obese rats (Culnan et al., 2010; Cummings et al., 2010; Ikezawa et al., 2012; Sun et al., 2013, 2014; Ramzy et al., 2014; Oh et al., 2016). Further, the feasibility, safety, and efficacy of the combination of IT surgery and sleeve gastrectomy in diabetic patients have been evaluated in several studies (De Paula et al., 2010; DePaula et al., 2012; Celik et al., 2015). It is a technically feasible operation and can be safely performed in T2DM patients with acceptable complication and mortality rates. Moreover, beyond glycemic control, other benefits such as lowering blood triglycerides and cholesterol and reducing hypertension have also been achieved in these patients. IT surgery has been speculated to regulate gut hormone secretion, alter host-microbial interactions and bile acid metabolism, and attenuate metabolic endotoxemia (Oh et al., 2016). However, the precise mechanisms underlying metabolic improvements are undetermined.

Fibroblast growth factor 21 (FGF21), a member of the fibroblast growth factor superfamily, is expressed and produced in the liver, pancreas, adipose tissues, and skeletal muscle with the liver being the major contributor to circulating FGF21 levels (Giralt et al., 2015). Hepatic FGF21 production is induced by peroxisome-proliferator-activated receptor-α (PPARα) (Giralt et al., 2015). FGF21 is regarded as an important and powerful metabolic regulator (Giralt et al., 2015). The primary physiological functions of FGF21 are involved in metabolic adaptations to fasting and ketogenic diets to promote gluconeogenesis, ketogenesis, and fatty acid oxidation (Giralt et al., 2015). FGF21 has also been reported to reduce blood glucose levels and improve insulin sensitivity under some pathological conditions, such as obesity and diabetes, by promoting thermogenesis and browning of white adipose tissue (WAT) (Giralt et al., 2015; So and Leung, 2016). FGF21 requires the co-receptor β-klotho (KLB) to activate the FGF receptor (FGFR) (Kilkenny and Rocheleau, 2016). There are several isoforms of FGFR (FGFR1-5), which are widely expressed in the body. Among the FGFRs, FGFR1 has the highest affinity for FGF21 and is mainly expressed in adipose tissue and pancreatic islets (Fisher and Maratos-Flier, 2016; Kilkenny and Rocheleau, 2016).

Although FGF21 plays a beneficial role in metabolism, circulating FGF21 levels have been reported to paradoxically increase in obesity and in T2DM patients and animal models, and they are positively correlated with the severity of glucose intolerance and insulin resistance, indicating an FGF21-resistant state (Chavez et al., 2009; Fisher et al., 2010; So et al., 2013; Roesch et al., 2015; Hu et al., 2016; So and Leung, 2016). It has been reported that the FGF21 signaling pathway is impaired in obese and diabetic animals, along with a decrease in its receptor or co-receptor in adipose tissue or pancreatic islets (Fisher et al., 2010; So et al., 2013; Nygaard et al., 2014; Gallego-Escuredo et al., 2015). In addition, FGF21 might be involved in the beneficial effects of bariatric surgery (Patton et al., 2017). However, the changes in circulating FGF21 levels after bariatric surgery have been discrepant. Circulating FGF21 levels have been reported to decrease after sleeve gastrectomy (SG) or gastric banding (GB), while they have remained unchanged, decreased or increased after Roux-en-Y gastric bypass (RYGB) in obese patients (Jansen et al., 2011; Haluzikova et al., 2013; Lips et al., 2014; Fjeldborg et al., 2017; Gomez-Ambrosi et al., 2017; Vienberg et al., 2017).

To the best of our knowledge, the effects of IT surgery on FGF21 remain unclear. Goto-Kakizaki (GK) rats, a non-obese Wistar substrain rat with spontaneous diabetes, have been reported to exhibit characteristics that are functionally present in human T2DM patients (Goto et al., 1975; Akash et al., 2013). GK rats have been widely used in research relating to IT surgery (Yan et al., 2012; Sun et al., 2013). Therefore, in the present study, experiments were conducted on GK rats to investigate the effects of IT surgery on glucose and lipid metabolism and the possible relationship of IT surgery with the FGF21 signaling pathway.

## Methods

### Animals

Ten-week-old male GK T2DM rats (weighing 279.2 ± 3.7 g) were purchased from National Rodent Laboratory Animal Resources (Shanghai, China). All of the rats were housed in standard cages in a temperature-controlled room (22–25°C) with humidity of ~60% and under a 12-h dark/light cycle. The rats were fed high fat diets (total energy: 45% fat, 35% carbohydrates, and 20% protein; H10045, Beijing HFK Bioscience Co. Ltd., Beijing, China) and water *ad libitum* before the operations were performed. After 1 week of acclimation, rats were randomly assigned to three groups (*n* = 7 per group): the IT group, Sham-IT group and no surgery group. All of the animal experimental protocols were performed according to the standards of the Guide for the Care and Use of Laboratory Animals. The protocols were approved by the ethics committee of Peking Union Medical College Hospital. All of the experimental protocols were performed according to the standards of the Laboratories-General Requirement for Biosafety (GB19489-2008).

### Surgical procedures

The surgical procedures were performed as described in our previous study (Chen et al., 2016). In brief, the rats were anesthetized with isoflurane (1.5–3%) after an overnight fasting. Then, ileal transposition was performed in rats from the IT group. A 10 cm ileal segment 5 cm proximal to the ileocecal valve was transected, transposed, and anastomosed isoperistaltically with the jejunum 5 cm distal to the Treitz ligament. The open ends of the ileal segments were anastomosed together using 7-0 silk sutures. Rats in the Sham-IT group underwent sham surgery, which involved the same incision, transection, and re-anastomosis of the gastrointestinal tract at multiple sites corresponding to the IT except for ileum transposition. The sham surgeries were prolonged to achieve similar operative times to those observed for the IT operations. The rats in the no surgery group did not receive any surgical intervention.

### Food intake, body weight, and fasting blood glucose levels

After surgery, the IT and Sham-IT rats were fed a non-residue diet (Ensure, Abbott Laboratories, Chicago, USA) for 1 week and then were fed a high-fat diet *ad libitum*. The no surgery rats were fed a high-fat diet *ad libitum* for the entire time. The food intake and body weights of the rats were measured three times per week for the first two postoperative weeks and weekly for the subsequent period. Fasting blood glucose was measured in blood collected from the tail tip using a glucometer (Roche One Touch® Ultra, Lifescan, Johnson & Johnson, Milpitas, USA) postoperatively at the 0, 1st, 2nd, 4th, and 6th weeks.

### Oral glucose tolerance test (OGTT) and intraperitoneal insulin tolerance test (IPITT)

The OGTT and IPITT were performed at the 6th week postoperatively. After fasting for 12 h, the rats were administered 2 g/kg glucose by oral gavage or 1 IU/kg insulin (Beijing SaiSheng Pharmaceutical Co. Ltd., Beijing, China) by intraperitoneal injection. The blood glucose levels were measured at 0, 30, 60, and 120 min after gavage or injection using the glucometer (Roche One Touch® Ultra, Lifescan, Johnson & Johnson, Milpitas, USA). The areas under the curve (AUCs) of the OGTT and IPITT were calculated by trapezoidal integration.

### Blood and tissue samples collection

Six weeks after surgery, the rats were administered an inhalation anesthesia using 2% isoflurane in an air/oxygen mixture after a 12 h of fasting. Blood samples were obtained from cardiac puncture and collected in chilled tubes containing a dipeptidyl peptidase IV inhibitor in EDTA solution. After centrifuging at 3,000 rpm and 4°C for 10 min, serum was immediately extracted and stored at −80°C. WAT, including epididymal adipose tissue (eWAT), perirenal adipose tissue (pWAT), and inguinal subcutaneous adipose tissue (sWAT), and brown adipose tissue (BAT) were dissected and weighted, respectively. BAT was obtained from the interscapular region. The WAT mass percentage was calculated by the percentage of total body weight occupied by the total WAT mass. Liver tissue (the left lateral liver lobe) was also collected. eWAT was bisected with one patch fixed in 10% formalin. The remaining eWAT and other tissue samples were immediately frozen in liquid nitrogen and stored at −80°C.

### Biochemical measurements

Serum fasting blood glucose (FBG), total cholesterol (TC), triglycerides (TG), low-density lipoprotein cholesterol (LDL-c), high-density lipoprotein cholesterol (HDL-c), free fatty acids (FFAs), lipoprotein(a) [Lp(a)], uric acid (UA), and high-sensitivity C reactive protein (hsCRP) were measured by routine automated laboratory methods. Serum insulin, fibroblast growth factor 21 (FGF21), glucagon-like peptide 1 (GLP-1), peptide YY (PYY), and leptin levels were measured by ELISA kits (CEA448Ra, CEC918Ra, CEA804Mi, CEB067Ra, and SEA084Ra, Wuhan USCN Business Co., Ltd., Wuhan, China) following the instructions of the manufacturers. The coefficients of variation for intraassay of insulin, FGF21, GLP1, PYY, and leptin were 2.9, 3.1, 2.0, 1.9, and 3.0%, respectively. Homeostasis model assessment of insulin resistance (HOMA-IR) was calculated according to the following formula: fasting serum insulin (pmol/L) × FBG (mmol/L)/135 (Yan et al., 2017).

### Histological analysis

After being fixed in 10% formalin, dehydrated and embedded in paraffin, eWAT was sliced into 5-μm sections and subjected to hematoxylin and eosin (H&E) staining. Slides were visualized under a 20 × objective of a light microscope (Nikon H550L, Japan), and images were obtained using a digital camera (Nikon DS-U3, Japan). The number of adipocytes was counted on five random fields per section at 200× magnification using microscopic image processing software (NIS element D, Japan), and the average cell number of every rat was calculated. The length and width of the field in the image processing software were measured to calculate the area. The adipocyte size of eWAT in the fixed area was calculated by the following formula: field area/adipocyte number (Jeong and Yoon, 2009).

### RT-qPCR analysis

RT-qPCR analysis was performed using SYBR Premix Ex Taq (Takara, Japan) and an ABI7500 PCR system (Applied Biosystems, San Francisco, CA, USA) as previously described (Yan et al., 2017). In brief, total RNA was extracted from adipose tissue using an RNeasy Lipid Tissue Mini Kit (Qiagen, Germany) and from liver tissue using an RNeasy Mini Kit (Qiagen, Germany) according to the supplier's instructions. One microgram of total RNA was reverse transcribed to cDNA using the PrimeScript™ RT reagent Kit with gDNA Eraser (TaKaRa, Japan). RT-qPCR was conducted in eWAT, pWAT, and sWAT to assess the expression of FGF21, FGFR1, KLB, and WAT browning-related genes, such as uncoupling protein 1 (UCP1), peroxisome proliferator-activated receptor gamma coactivator 1α (PGC1α), PR domain containing 16 (PRDM16), transmembrane protein 26 (Tmem26), cell death-inducing DNA fragmentation factor alpha subunit-like effector A (CIDEA), and cytochrome C oxidase subunit VIIIb (Cox8b). RT-qPCR was also conducted in the liver to assess the expression of FGF21, glycogen synthase 2 (GYS2), PPARα, tumor necrosis factor α (TNFα), and interleukin-6 (IL-6). β-actin was used for normalization and the relative expression of each target gene was calculated using the formula 2^−ΔΔ*Ct*^. RT-qPCR was duplicated for 2 wells in a final volume of 20 μL. The primers used to amplify the target genes and β-actin were listed in the Table S1.

### Western blot analysis

Total proteins were extracted from adipose tissue and liver tissue using a Total Protein Extraction kit (Applygen, Beijing, China) according to the manufacturer's instructions. Thirty micrograms of extracted proteins were separated by 10% SDS-PAGE and transferred onto nitrocellulose membranes (Millipore, USA) using wet transfer (BIO-RAD, CA, USA). The membranes were blocked with 5% non-fat milk (BD, NJ, USA) diluted with 1 × TBST for 2 h, and then they were incubated with primary antibodies at 4°C overnight. Rabbit immunoglobulin G (IgG) anti-β actin (CST, 4970, USA), anti-FGFR1 (CST, 9740, USA), anti-glycogen synthase (CST, 3886, USA), anti-phospho-glycogen synthase (Ser641) (CST, 3891, USA), anti-FGF21 (Abcam, ab171941, Cambridge, UK), anti-UCP1 (Abcam, ab10983, Cambridge, UK), and anti-KLB (LSBio, LS-B3568, USA) were used as the primary antibodies (1:1,000–1:2,000 diluted in 5% non-fat milk). The membranes were then incubated with HRP-conjugated secondary antibody (ZSGB-BIO, Beijing, China) at room temperature for 1 h. Protein bands were visualized using the SuperSignal West Pico Chemiluminescent Substrate (Pierce, Rockford, USA) according to the manufacturer's instructions. Specific bands were determined according to the molecular weight of the target protein and quantified using ImageJ software (version 1.48, National Institutes of Health, Bethesda, MD, USA). β-actin was used as an internal reference protein, and the density ratio of the target proteins to β-actin was calculated to evaluate the protein expression differences. The relative expression of target proteins was further normalized by considering the average of the no surgery group to be equal to 1.

### Glycogen content measurements in liver tissue from GK rats

Liver glycogen content was measured using a Liver Glycogen Assay Kit (Nanjing Jiancheng Bioengineering Institute, Nanjing, China) following the manufacturer's instructions. Briefly, rat liver tissues were placed in concentrated alkaline solution and boiled for 30 min to destruct other constituents but to keep the glycogen. Then, the liver glycogen was dehydrated by concentrated sulfuric acid to produce furfural derivatives, which reacted with anthrone to generate a blue color at an OD of 620 nm. The light absorption value was detected by UV1600 (SHIMADZU, Japan). The liver glycogen content was calculated according to the standard curve and was normalized by liver tissue weight.

### Statistical analysis

Results are expressed as the mean ± standard error of measurement (SEM) or the median (interquartile range). The univariate analysis of variance (ANOVA) method was used for data analysis. Dunnett's multiple comparisons test was used for two group comparisons depending on the homogeneity of variance. Skewed data were ln-transformed, and the Kruskal–Wallis test was used if the data were still not normally distributed. All of the statistical computations were run using SPSS software, version 22.0 for Windows (SPSS Inc., Chicago, IL, USA), and *P* < 0.05 was considered to be statistically significant.

## Results

### Effects of IT surgery on body weight and food intake in diabetic GK rats

**Figure 1:**
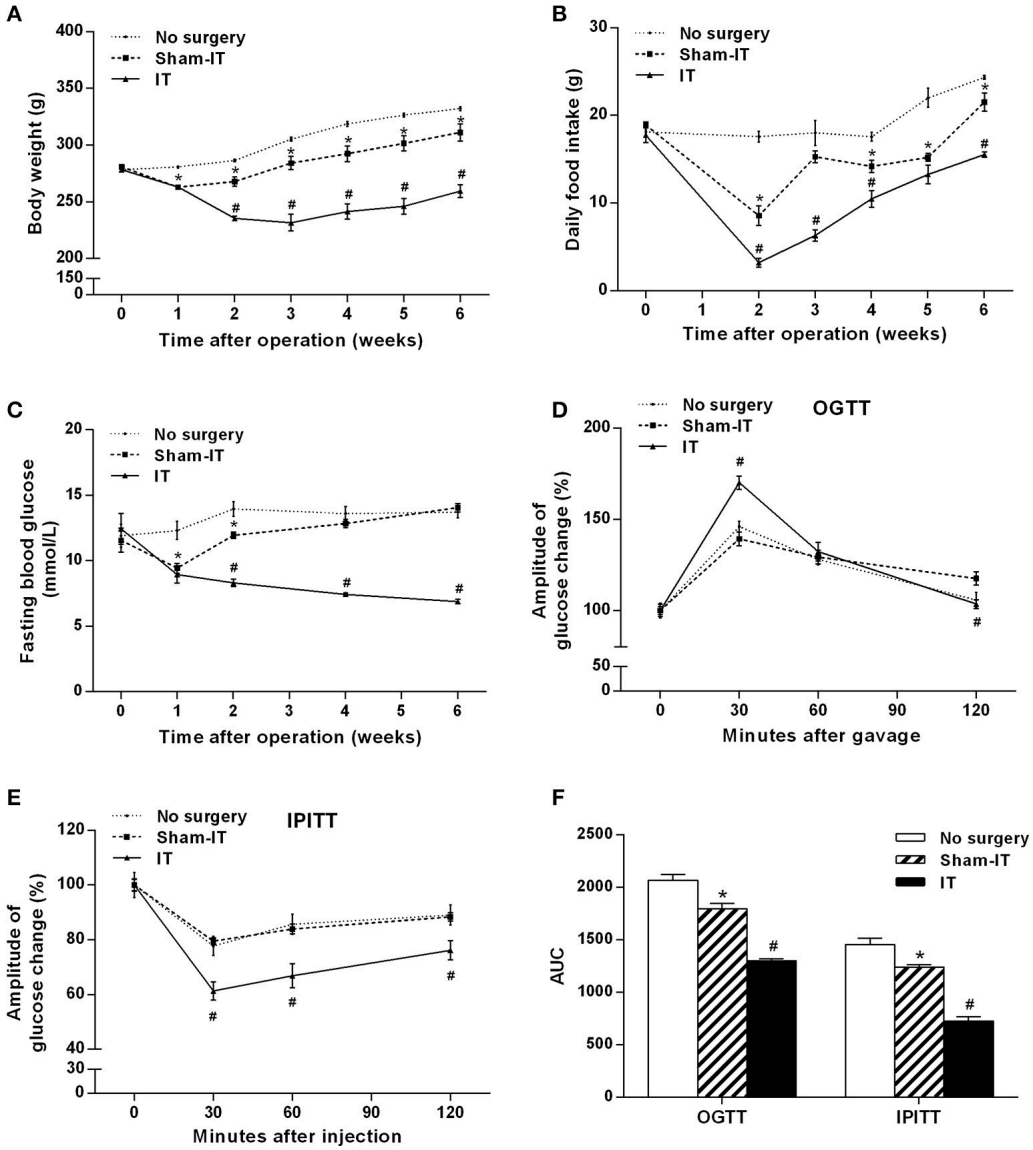
Effects of IT surgery on the body weight, food intake, glucose metabolism and insulin sensitivity of diabetic GK rats. GK rats underwent IT or Sham-IT surgery, and the changes in body weight **(A)**, food intake **(B)**, and fasting blood glucose levels **(C)** were observed 6 weeks after IT surgery. The oral glucose tolerance test (OGTT) and intraperitoneal insulin tolerance test (IPITT) were performed in the rats as described in the “Methods” section. Glucose levels at 0 (before gavage or injection), 30, 60, and 120 min were measured and normalized to T0 (100%) to present the amplitude of glucose changes by the OGTT **(D)** and IPITT **(E)**. Areas under the curve (AUCs) from the OGTT and IPITT were calculated as described in the “Materials and Methods” section **(F)**. The data are represented as the mean ± SEM. *P < 0.05 vs. the No surgery group, ^#^*P* < 0.05 vs. the Sham-IT group. (*n* = 7 in the No surgery group, *n* = 6 in the Sham-IT group, and *n* = 5 in the IT group).

The IT and Sham-IT surgeries were all smoothly performed. After surgery, two IT rats and one Sham-IT rat died due to intestinal obstruction on the 6th, 12th, and 14th days after surgery. No other severe complications of surgery were observed. Before surgery, there was no significant difference in body weight and food intake among the three groups. As shown in Figures 1A,B, the body weight and food intake of the IT rats were significantly decreased by 12.2 and 62.6%, respectively, at the beginning of the 2nd week after surgery compared with the Sham-IT rats (all *P* < 0.05), and this decrease continued to the 6th week, when the body weight and food intake of the IT rats were 83.4 and 72.2% of those of the Sham-IT rats, respectively (all *P* < 0.05), while the body weight and food intake of the Sham-IT rats also decreased by 6.5 and 11.6%, respectively, at the 6th week after surgery, compared with the no surgery rats (all *P* < 0.05).

### Effects of IT surgery on glucose and lipid metabolism and insulin sensitivity in diabetic GK rats

The rats in the IT group showed significant improvement in glucose metabolism and insulin sensitivity. As presented in Figure 1C, the FBG levels of the IT rats decreased from the initial 12.4 ± 1.1 mmol/L to 6.9 ± 0.1 mmol/L at the 6th week post-surgery, and the FBG levels were always remarkably lower than those of the Sham-IT rats from the 2nd week to 6th week post-surgery (all *P* < 0.05). As shown in Figure 1D, blood glucose decreased more rapidly in the IT group after the initial rise at the 30 min time point in the OGTT. In the IPITT, blood glucose also decreased more rapidly, and the amplitude of the blood glucose decrease was greater in the IT group following insulin injection (Figure 1E, *P* < 0.05). Raw data of OGTT and IPITT were presented in Table S2. The AUCs of the OGTT and IPITT in the IT rats decreased by 27.6 and 41.5%, respectively, compared with the Sham-IT rats (Figure 1F, *P* < 0.05). The HOMA-IR of the IT rats was also decreased compared with the Sham-IT rats, as displayed in Table 1 (*P* < 0.05). While the AUCs of the OGTT and IPITT in the Sham-IT rats also showed slight reductions by 13.2 and 14.7%, respectively (Figure 1F, *P* < 0.05), the FBG levels at the 6th week and the HOMA-IR were not different between the no surgery and Sham-IT groups (Figure 1C, Table 1). In addition, there were no significant differences in the levels of serum lipid profiles or hsCRP among the three groups as shown in Table 1.

**Table 1:** Effects of IT surgery on serum levels of biochemical parameters, insulin, FGF21, leptin, and gastrointestinal hormones in diabetic GK rats^Δ^.

	**No surgery (*n* = 7)**	**Sham-IT (*n* = 6)**	**IT (*n* = 5)**
TC (mmol/L)	2.48 ± 0.08	1.97 ± 0.26	2.22 ± 0.18
TG (mmol/L)	0.48 ± 0.07	0.57 ± 0.16	0.67 ± 0.10
LDL-c (mmol/L)	0.23 ± 0.01	0.23 ± 0.02	0.22 ± 0.02
HDL-c (mmol/L)	0.71 ± 0.03	0.53 ± 0.09	0.63 ± 0.05
FFA (μmol/L)	319.70 (200.10, 356.60)	463.55 (366.75, 505.75)^*^	374.70 (342.60, 556.95)
Lp(a) (mg/L)	2.40 (0.70, 3.00)	2.55 (1.13, 3.28)	2.70 (1.05, 3.55)
UA (μmol/L)	106.21 ± 10.15	113.68 ± 9.87	125.18 ± 14.56
hsCRP (mg/L)	0.05 ± 0.01	0.06 ± 0.01	0.05 ± 0.01
Insulin (ng/mL)	1.95 (1.81, 2.13)	1.76 (1.53, 2.21)	1.22 (0.85, 2.05)
HOMA-IR	34.86 (30.29, 38.59)	30.55 (26.32, 39.15)	11.01 (7.50, 16.50)^#^
FGF21 (pg/mL)	460.01 ± 27.52	443.45 ± 15.64	327.03 ± 24.31^#^
Leptin (ng/mL)	0.99 ± 0.15	1.49 ± 0.47	0.57 ± 0.18^#^
GLP-1 (pg/mL)	16.41 ± 0.6	16.22 ± 0.49	15.73 ± 0.45
PYY (pg/mL)	476.45 ± 43.56	604.11 ± 36.98	527.29 ± 60.18

Δ Values are the mean ± SEM or the median (25th−75th %). TC, total cholesterol; TG, triglycerides; LDL-c, low-density lipoprotein cholesterol; HDL-c, high-density lipoprotein cholesterol; FFA, free fatty acid; Lp(a), lipoprotein(a); UA, uric acid; hsCRP, high-sensitivity C reactive protein; HOMA-IR, homeostasis model assessment of insulin resistance; FGF21, fibroblast growth factor 21; GLP-1, glucagon like peptide 1; PYY, peptide YY.

* P < 0.05 vs. the No surgery group,

# *P < 0.05 vs. the Sham-IT group*.

### Effects of IT surgery on serum levels of FGF21, leptin, and gastrointestinal hormones in diabetic GK rats

Serum levels of FGF21, leptin, GLP-1, and PYY of all of the rats were measured 6 weeks after IT surgery. As presented in Table 1, serum FGF21 levels of IT rats were notably decreased by 26.3%, compared with that of the Sham-IT rats (*P* < 0.05), while the serum leptin levels of the IT rats also showed a 61.7% reduction, compared with the Sham-IT rats (*P* < 0.05). However, there were no significant differences in serum levels of GLP-1 and PYY among the three groups.

### Effects of IT surgery on fat mass and adipocyte size in diabetic GK rats

**Figure 2:**
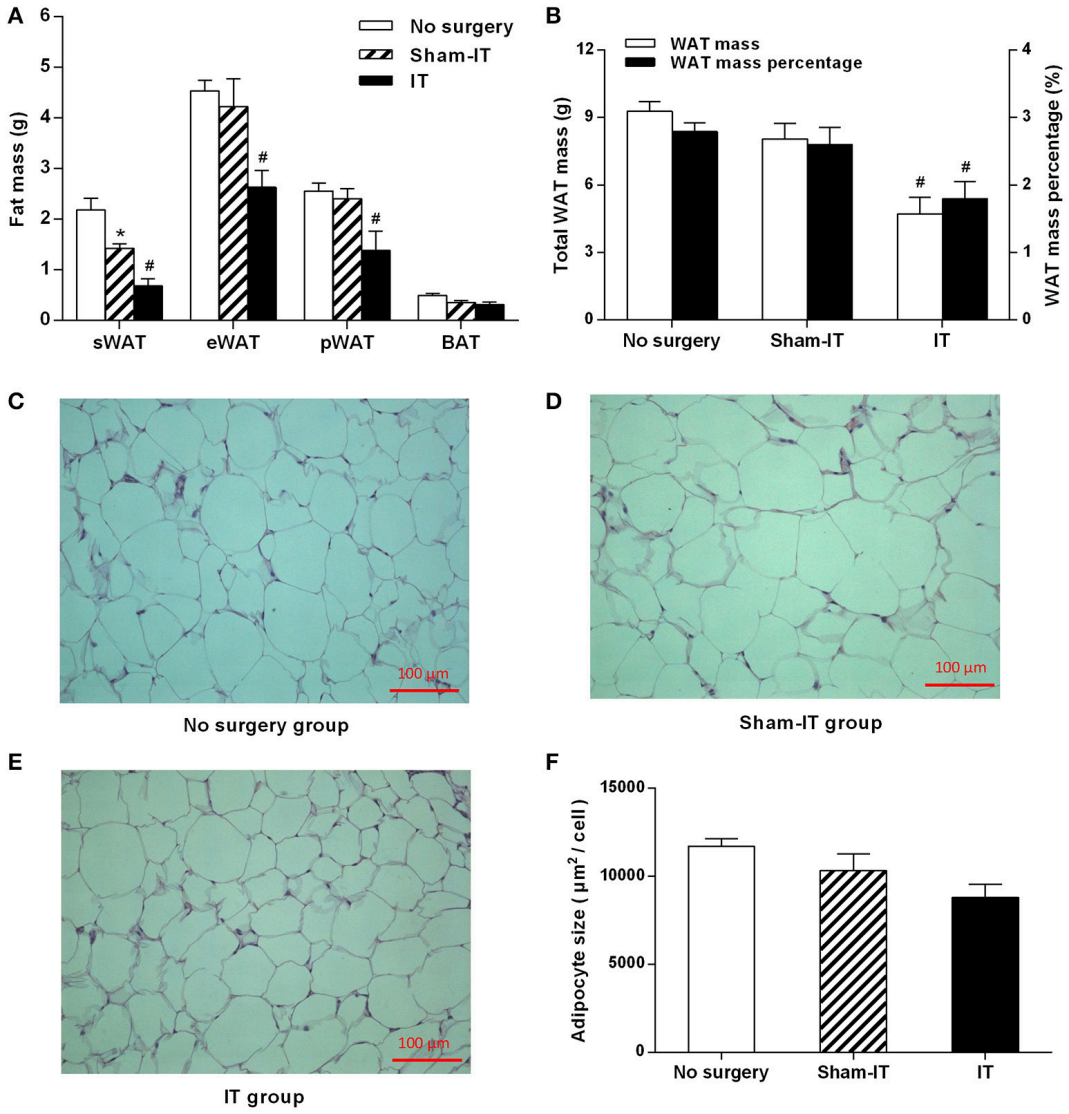
Effects of IT surgery on fat mass and the adipocyte size of the eWAT in diabetic GK rats. GK rats were underwent IT or Sham-IT surgery. Six weeks after surgery, white adipose tissue (WAT), including sWAT, eWAT, and pWAT, and brown adipose tissue (BAT) were obtained from the rats and weighed **(A)**. The WAT mass percentage was calculated by the percentage of body weight occupied by the total WAT mass **(B)**. Representative H&E staining of eWAT sections from GK rats in the No surgery **(C)**, Sham-IT **(D)**, and IT groups **(E)** at 200 × magnification (scale bars, 100 μm). The adipocyte size of the eWAT of GK rats in the No surgery, Sham-IT, and IT groups were calculated as described in the “Methods” section **(F)**. The data are represented as the mean ± SEM. *P < 0.05 vs. the No surgery group, ^#^*P* < 0.05 vs. the Sham-IT group (*n* = 7 in the No surgery group, *n* = 6 in the Sham-IT group, and *n* = 5 in the IT group).

Six weeks after surgery, the masses of the sWAT, eWAT, and pWAT of the IT rats were decreased by 0.74, 1.59, and 1.01 g, respectively, compared with those of the Sham-IT rats (Figure 2A, *P* < 0.05), and the total WAT mass and WAT mass percentage of the IT rats were notably decreased by 41.5 and 30.9%, respectively (Figure 2B, *P* < 0.05). In addition, the adipocyte size in the eWAT of the IT rats tended to be smaller than that of the Sham-IT rats under the light microscope, as presented in Figures 2C–E, although no significant difference was observed, as shown in Figure 2F (*P* = 0.16). The sWAT mass of the Sham-IT rats also presented a 34.5% reduction, compared with the no surgery rats (Figure 2A, *P* < 0.05). However, there was no significant change in BAT mass among the three groups.

### Effects of IT surgery on the FGF21 signaling pathway and WAT browning-related genes in the eWAT of diabetic GK rats

**Figure 3:**
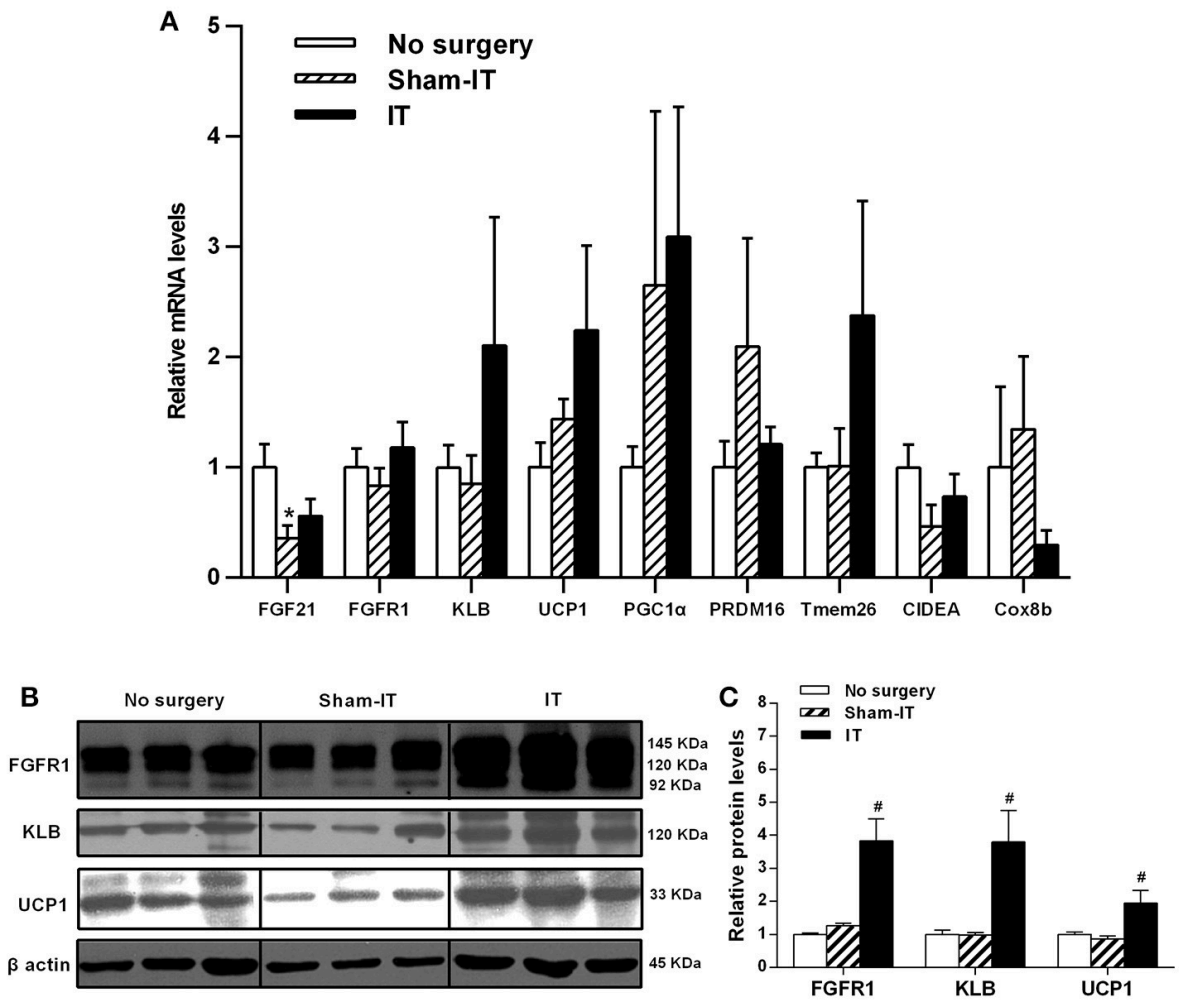
Effects of IT surgery on the FGF21 signaling pathway and WAT browning-related genes in the eWAT of diabetic GK rats. The mRNA levels of FGF21, its receptor FGFR1 and the co-receptor KLB and WAT browning-related genes (UCP1, PGC1α, PRDM16, Tmem26, CIDEA, and Cox8b) in the eWAT of GK rats in the No surgery, Sham-IT and IT groups were determined by RT-qPCR analysis **(A)**. The protein levels of FGFR1, KLB, and UCP1 in the eWAT of GK rats were determined by western blot analysis (**B**, *n* = 3 in representative blots from each group), and the density ratios of these proteins to β-actin were calculated to evaluate the protein expression differences **(C)**. The data are represented as the mean ± SEM. *P < 0.05 vs. the No surgery group, ^#^*P* < 0.05 vs. the Sham-IT group (*n* = 7 in the No surgery group, *n* = 6 in the Sham-IT group, and *n* = 5 in the IT group).

As presented in Figure 3, although the mRNA levels of FGF21, FGFR1, and its co-receptor KLB in eWAT were not different between the IT and Sham -IT rats, the protein levels of both FGFR1 and KLB of IT rats notably elevated to 3.0- and 3.9-fold of that of the Sham-IT rats, respectively (all *P* < 0.05). Further investigation showed that, while the mRNA levels of WAT browning-related genes, including UCP1, PGC1α, PRDM16, Tmem26, CIDEA, and Cox8b, in eWAT were not different between the IT and Sham-IT rats (Figure 3A), the UCP1 protein levels of the IT rats significantly increased to 2.2-fold of that of the Sham-IT rats (Figures 3B,C, *P* < 0.05).

### Effects of IT surgery on the FGF21 signaling pathway and WAT browning-related genes in the pWAT of diabetic GK rats

**Figure 4:**
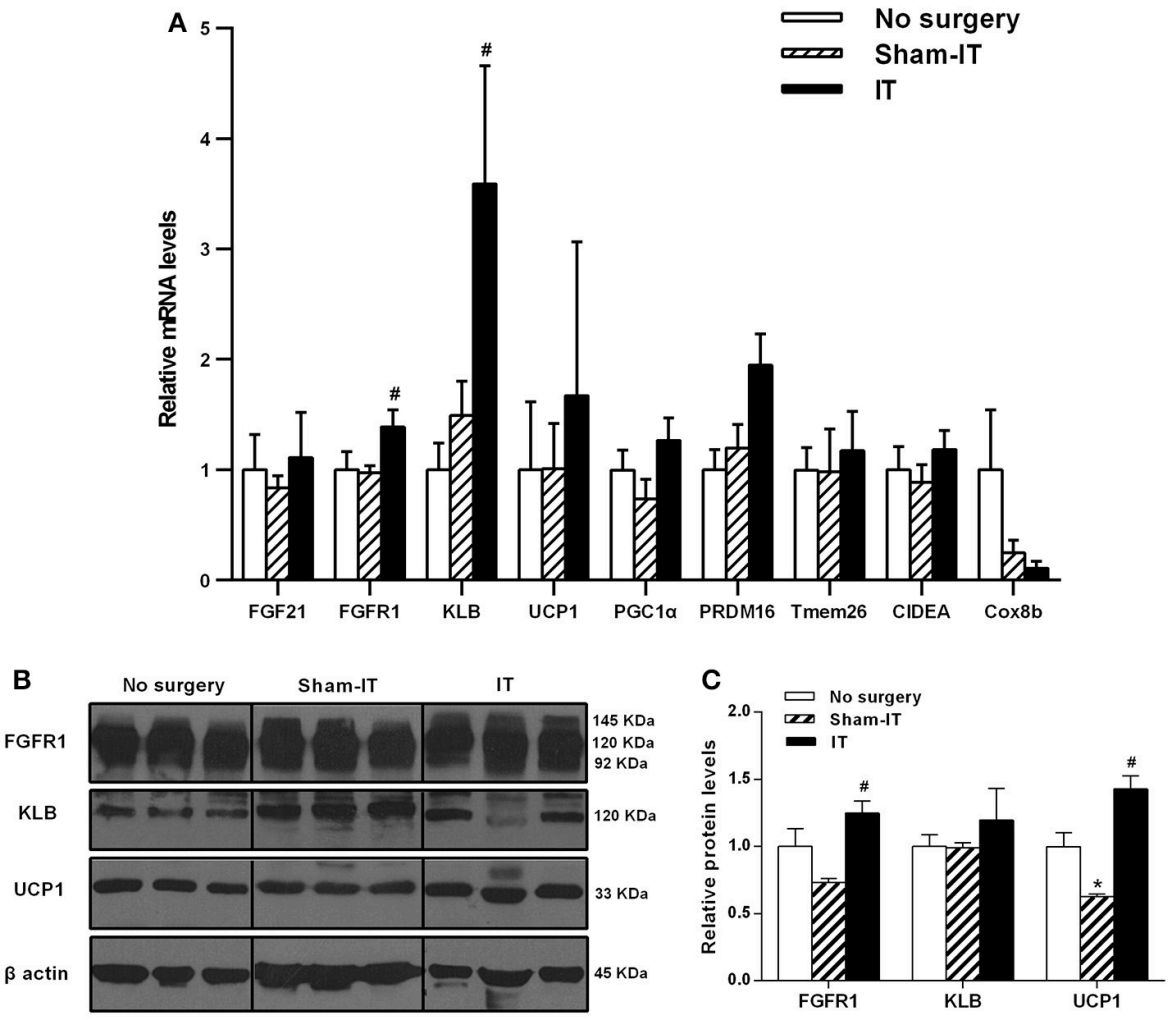
Effects of IT surgery on the FGF21 signaling pathway and WAT browning-related genes in the pWAT of diabetic GK rats. The mRNA levels of FGF21, its receptor FGFR1 and the co-receptor KLB and WAT browning-related genes (UCP1, PGC1α, PRDM16, Tmem26, CIDEA, and Cox8b) in the pWAT of GK rats in the No surgery, Sham-IT and IT groups were determined by RT-qPCR analysis **(A)**. The protein levels of FGFR1, KLB and UCP1 in the pWAT of GK rats were determined by western blot analysis (**B**, *n* = 3 in representative blots from each group), and the density ratios of these proteins to β-actin were calculated to evaluate the protein expression differences **(C)**. The data are represented as the mean ± SEM. *P < 0.05 vs. the No surgery group, ^#^*P* < 0.05 vs. the Sham-IT group (*n* = 7 in the No surgery group, *n* = 6 in the Sham-IT group, and *n* = 5 in the IT group).

As shown in Figure 4A, the mRNA levels of FGFR1 and KLB in the pWAT of the IT rats were increased to 1.4- and 2.4-fold of that in the Sham-IT rats (*P* < 0.05). Similar to eWAT, the protein levels of FGFR1 and UCP1 of the IT rats were significantly increased to 1.7- and 2.3-fold of that of the Sham-IT rats, respectively (Figures 4B,C, *P* < 0.05). In addition, there were no significant differences in the mRNA levels of WAT browning-related genes in pWAT between the IT and Sham-IT groups (Figure 4A). Compared with the no surgery group, the UCP1 protein levels in Sham-IT group were notably reduced (Figure 4C, *P* < 0.05).

### Effects of IT surgery on the FGF21 signaling pathway and WAT browning-related genes in the sWAT of diabetic GK rats

**Figure 5:**
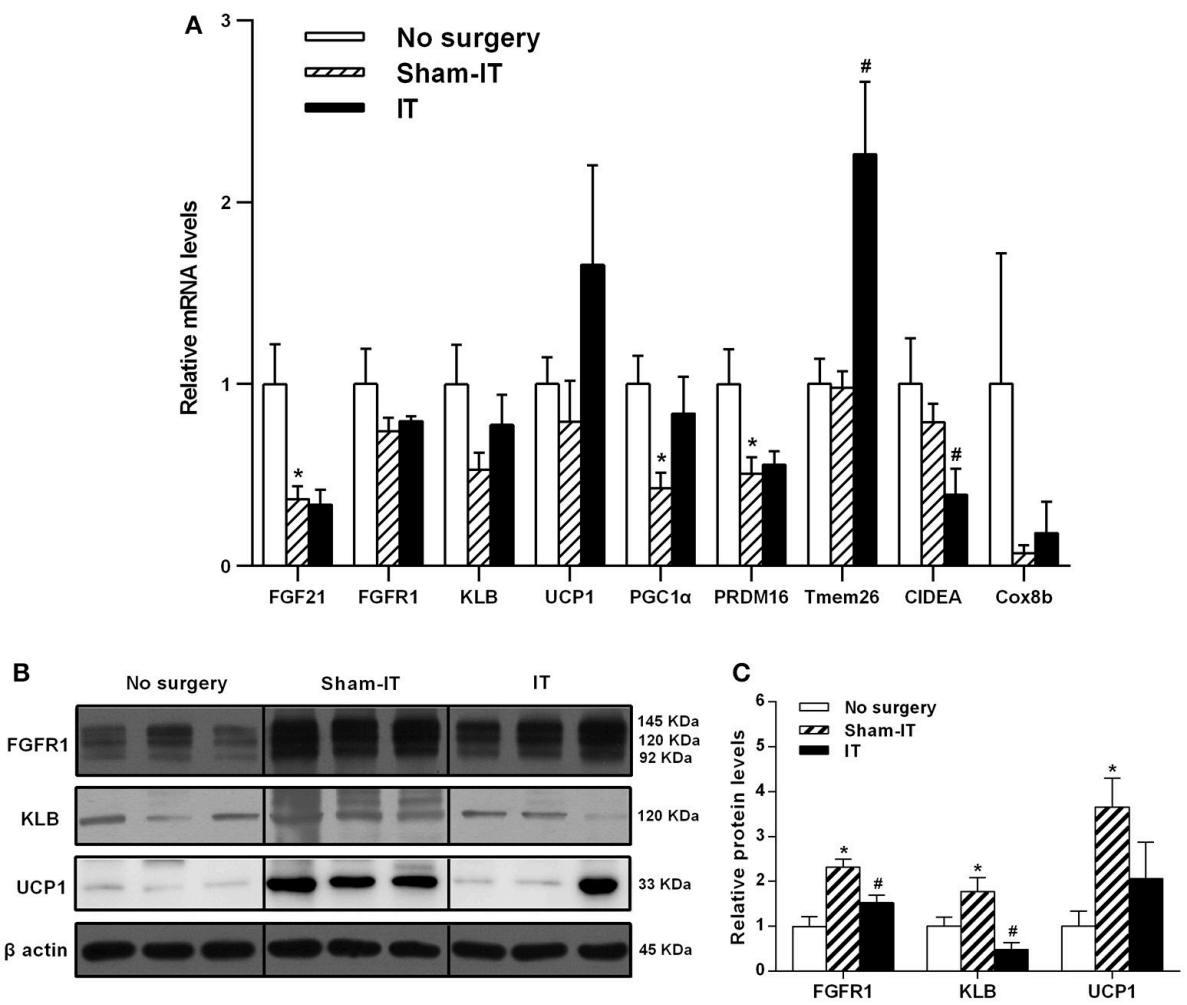
Effects of IT surgery on the FGF21 signaling pathway and WAT browning-related genes in the sWAT of diabetic GK rats. The mRNA levels of FGF21, its receptor FGFR1 and the co-receptor KLB and WAT browning-related genes (UCP1, PGC1α, PRDM16, Tmem26, CIDEA, and Cox8b) in the sWAT of GK rats in the No surgery, Sham-IT and IT groups were determined by RT-qPCR analysis **(A)**. The protein levels of FGFR1, KLB, and UCP1 in the sWAT of GK rats were determined by western blot analysis (**B**, *n* = 3 in representative blots from each group) and the density ratios of these proteins to β-actin were calculated to evaluate the protein expression differences **(C)**. The data are represented as the mean ± SEM. *P < 0.05 vs. the No surgery group, ^#^*P* < 0.05 vs. the Sham-IT group (*n* = 7 in the No surgery group, *n* = 6 in the Sham-IT group, and *n* = 5 in the IT group).

Regarding sWAT, Tmem26 mRNA levels in the sWAT of the IT rats significantly increased, while CIDEA significantly decreased compared with the Sham-IT rats (Figure 5A, *P* < 0.05). At the same time, the protein levels of both FGFR1 and KLB in the sWAT of the IT rats were notably decreased by 34.4 and 72.1%, respectively, compared with the Sham-IT rats (Figures 5B,C, *P* < 0.05), and the UCP1 protein levels also tended to decrease (Figures 5B,C, *P* = 0.084). In addition, the protein levels of FGFR1, KLB and UCP1 in sWAT of the Sham-IT rats were increased to 2.3-, 1.7-, and 3.7-fold of those of the no surgery rats (Figures 5B,C, *P* < 0.05), while the mRNA levels of FGF21, PGC1α, and PRDM16 were significantly decreased, compared with those of the no surgery rats (Figure 5A, *P* < 0.05).

### Effects of IT surgery on the FGF21 signaling pathway, glycogen synthesis, and the expression of PPARα and inflammatory factors in the livers of diabetic GK rats

**Figure 6:**
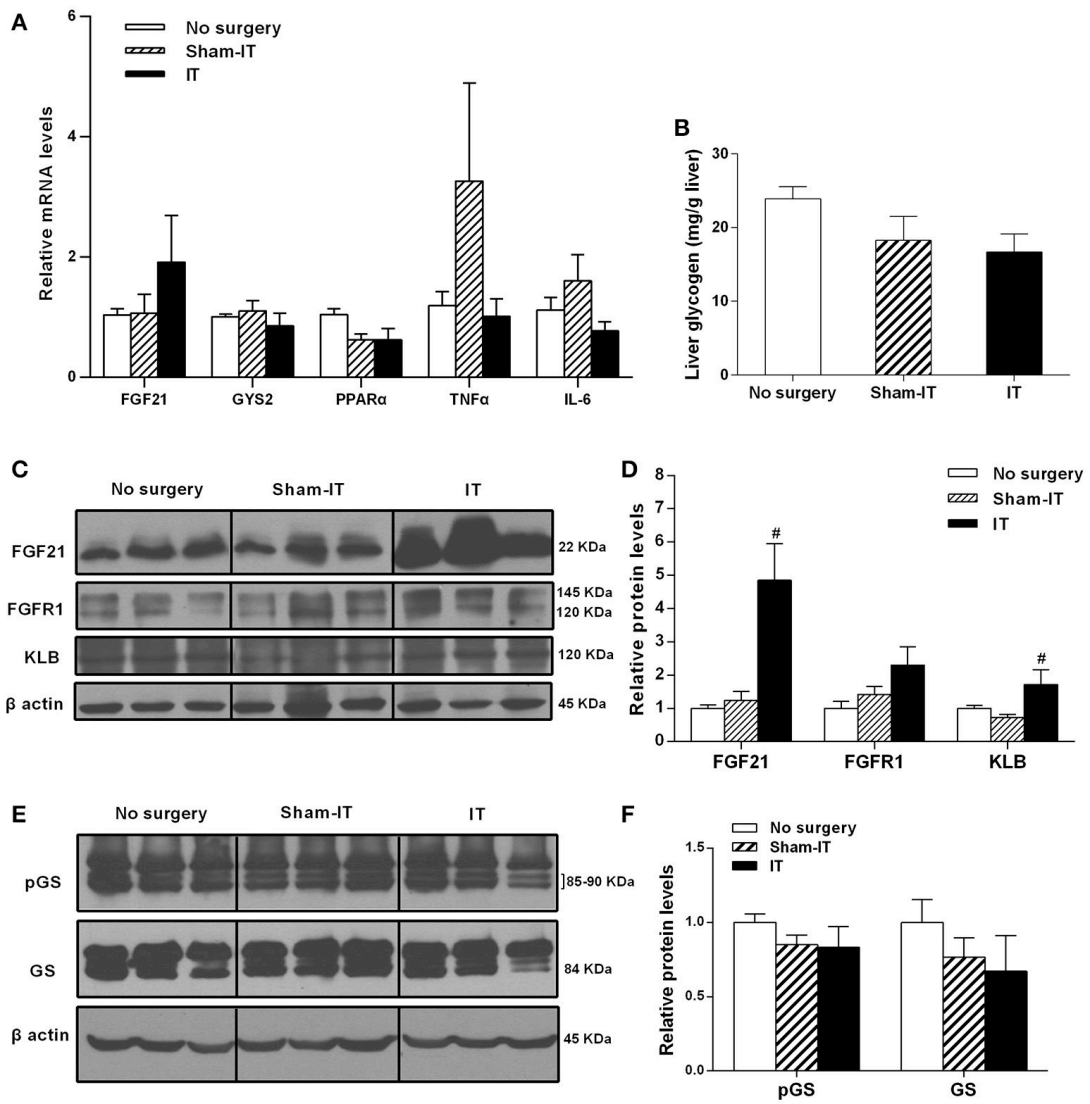
Effects of IT surgery on the FGF21 signaling pathway, glycogen synthesis, and the expression of PPARα and inflammatory factors in the livers of diabetic GK rats. The mRNA levels of FGF21, GYS2, PPARα, and the inflammatory cytokines TNFα and IL-6 in the livers of GK rats in the No surgery, Sham-IT, and IT groups were determined by RT-qPCR analysis **(A)**. Liver glycogen content of GK rats in these three groups were determined by the glycogen assay kit as described in the “Methods” section **(B)**. The protein levels of FGF21, its receptor FGFR1 and the co-receptor KLB, glycogen synthase (GS) and its phosphorylated form (pGS) in the livers of GK rats were determined by western blot analysis (**C,E**, *n* = 3 in representative blots from each group), and the density ratios of these proteins to β-actin were calculated to evaluate the protein expression differences **(D,F)**. The data are represented as the mean ± SEM. ^#^*P* < 0.05 vs. the Sham-IT group (*n* = 7 in the No surgery group, *n* = 6 in the Sham-IT group, and *n* = 5 in the IT group).

As shown in Figure 6, although the FGF21 mRNA levels in liver tissue were not different between the IT and Sham-IT rats, the protein levels of FGF21 and KLB in the IT rats were significantly increased to 3.9- and 2.3-fold of those of the Sham-IT rats, respectively (*P* < 0.05). Further investigations were performed to explore the effects of IT surgery on the factors regulating hepatic FGF21 production and post-receptor events after the activation of the FGF21 signaling pathway in liver. As a result, there were no changes in the mRNA levels of PPARα, the upstream regulating factor of hepatic FGF21 production (Figure 6A). There were also no differences in the mRNA levels of TNFα, IL-6, and GYS2 (Figure 6A), liver glycogen content (Figure 6B), and the protein levels of glycogen synthase and its phosphorylation (Figures 6E,F) between the IT and Sham-IT groups.

## Discussion

IT surgery, as a bariatric and metabolic surgery, has been reported to be an innovative metabolic operation with many beneficial effects for metabolic improvement (Cohen et al., 2016; Oh et al., 2016). In the current study, we demonstrated that IT surgery could significantly reduce body weight, decrease FBG levels and increase insulin sensitivity in diabetic GK rats. Consistent with our findings, the beneficial effects of IT surgery have also been reported by several researchers, who demonstrated that IT surgery significantly decreased body weight, food intake, and FBG levels and reduced the oral glucose tolerance and insulin resistance of GK rats or other diabetic rats, such as Otsuka Long-Evans Tokushima Fatty (OLETF) rats, University of California at Davis type 2 diabetes mellitus (UCD-T2DM) rats, Zucker or high fat diet (HFD)-induced obese rats and Sprague Dawley (SD) rats (Culnan et al., 2010; Cummings et al., 2010; Kohli et al., 2010; Gaitonde et al., 2012; Ikezawa et al., 2012; Yan et al., 2012; Nausheen et al., 2013; Sun et al., 2013, 2014; Ramzy et al., 2014). Additionally, IT surgery has also been reported to significantly reduce fat mass in diabetic rats (Cummings et al., 2010; Ikezawa et al., 2012). A study conducted by Ikezawa et al demonstrated that IT surgery notably reduced the eWAT mass of OLETF diabetic rats (Ikezawa et al., 2012), and a study performed by Cummings et al also showed a significant reduction in visceral fat mass, including eWAT and pWAT, in UCD-T2DM rats after IT surgery (Cummings et al., 2010). In our present study, there were remarkable decreases in both subcutaneous and visceral fat mass after IT surgery. Taken together, all of these results demonstrated that IT surgery could reduce body weight, fat mass and food intake, improve glucose metabolism, and increase insulin sensitivity.

FGF21 has been considered a powerful metabolic regulator related to glucose and lipid metabolism and energy homeostasis (Gimeno and Moller, 2014; Giralt et al., 2015). In non-diabetic Wistar rats, circulating FGF21 levels is reported in the range of 90–300 pg/mL (Asrih et al., 2016; Benomar et al., 2016; Charoenphandhu et al., 2017). Meanwhile, GK rats in our study had FGF21 levels between 320 and 460 pg/mL, which is consistent with the elevated levels observed in individuals with obesity and diabetes, and in other animal models of diabetes (Chavez et al., 2009; Nygaard et al., 2014; Gallego-Escuredo et al., 2015; Roesch et al., 2015; Hu et al., 2016). Furthermore, in adipose tissue, others have reported decreased expression of the FGF21 receptor and/or its co-receptor KLB in diabetes and obesity, suggesting a state of FGF21 resistance (Fisher et al., 2010; Nygaard et al., 2014; Gallego-Escuredo et al., 2015; So and Leung, 2016). Circulating FGF21 levels, however, can be reduced after certain surgical or medical interventions. For example, serum FGF21 levels were notably reduced after SG or GB surgery in obese patients (Haluzikova et al., 2013; Lips et al., 2014; Gomez-Ambrosi et al., 2017) or after RYGB surgery in obese patients or high fat diet-induced obese rats (Mosinski et al., 2016; Fjeldborg et al., 2017). There was also a remarkable decrease in plasma FGF21 levels after treatment with some drugs, such as exenatide, metformin, or mitiglinide, in new-onset T2DM patients (Samson et al., 2011; Wang et al., 2012; Fan et al., 2016; Hu et al., 2016). The reduction of circulating FGF21 has been considered a sign of improvement in FGF21 resistance (Samson et al., 2011; Hu et al., 2016). Nevertheless, to the best of our knowledge, there are currently no reports concerning FGF21 level changes after IT surgery. In the present study, we found for the first time that serum FGF21 levels of diabetic GK rats displayed significant decreases 6 weeks after IT surgery. To further explore whether the notable decrease of serum FGF21 in rats after IT surgery came from the alleviation of FGF21 resistance, the changes in the FGF21 signaling pathway in adipose tissue and the liver were investigated in both the mRNA and protein levels. Adipose tissue has been considered the key target tissue of FGF21 (Gimeno and Moller, 2014; Giralt et al., 2015), and FGF21 has been reported to promote WAT browning (Fisher and Maratos-Flier, 2016). Therefore, the expression levels of FGFR1, its co-receptor KLB and WAT browning-related genes in WAT were determined. Interestingly, the protein levels of FGFR1 and KLB in the visceral WAT of GK rats were first found to be notably increased after IT surgery, and UCP1, a WAT browning marker, was also found to be significantly increased in the current study. In accordance with our results, Neinast et al reported that UCP1 expression in the gonadal adipose tissue of mice was significantly increased after RYGB (Neinast et al., 2015). Studies conducted by Ikezawa et al also demonstrated that IT surgery increased UCP1 protein expression in the BAT of diabetic rats (Ikezawa et al., 2012). All of these findings, together with our results, suggest that IT surgery might strengthen the FGF21 signaling pathway to improve FGF21 resistance by promoting WAT browning in diabetic rats.

In contrast to the observations in visceral WAT, the expression levels of FGFR1, KLB, and CIDEA in the sWAT of rats that underwent IT surgery were significantly decreased, and the UCP1 protein levels also tended to decrease. The reasons for the opposite effects of IT surgery on visceral WAT and sWAT must be further investigated. One possible explanation might be the different biological functions of visceral adipose tissue (VAT) and subcutaneous adipose tissue (SAT) in the development of metabolic disorders. It is well-known that VAT is more metabolically active, more sensitive to lipolysis, and more insulin resistant than SAT (Ibrahim, 2010). In the present study, because the total VAT mass was ~3.0-fold higher than the SAT mass, the improvement of VAT function, such as increased WAT browning, could explain the improvement of whole body glucose metabolism in diabetic GK rats after IT surgery. Similarly, it was reported by Yang et al. that browning of VAT could improve global glucose and lipid metabolism, insulin sensitivity and hepatic steatosis in HFD-induced obese mice (Yang et al., 2017).

There was also a significant increase in the expression of FGF21 and its co-receptor KLB in the livers of rats after IT surgery, suggesting that the hepatic FGF21 signaling pathway might also improve after IT surgery. Thus, further investigation of the aspects of FGF21 production and its downstream signaling pathway in the liver was performed in the present study. It has been documented in some studies that hepatic FGF21 production is primarily induced by PPARα (Giralt et al., 2015), and FGF21 is also an autocrine factor of the liver that could promote liver glycogen synthesis (Gong et al., 2016). Moreover, it has been reported that FGF21 could suppress the expression of inflammatory cytokines, such as TNFα and IL-6 (Lee et al., 2012; Wang et al., 2015; Patton et al., 2017). Nevertheless, there were no changes in the expression of PPARα, TNFα, IL-6, and glycogen synthase and glycogen contents in the livers of rats after IT surgery. These results suggested that IT surgery increased FGF21 production and the co-receptor KLB in the liver, while the effects might not relate to PPARα-induced FGF21 production, increased glycogen synthesis or reduced inflammatory cytokine expression. However, the lack of significant differences in these gene expressions levels might be due to high interindividual variability, especially in the IT group, which had only five rats. Therefore, the effects of IT surgery on the liver in relation to the FGF21 signaling pathway activation remain to be further explored in the future.

Finally, leptin is a hormone mainly secreted by adipose tissue, and the primary function of leptin is to regulate fat stores (Harris, 2014). In the present study, serum leptin levels significantly decreased after IT surgery. Consistent with our result, it has been demonstrated in previous studies that plasma leptin concentrations were significantly decreased after IT surgery in diabetic or non-diabetic rats (Kohli et al., 2010; Ikezawa et al., 2012; Nausheen et al., 2013; Ramzy et al., 2014). The decrease in circulating leptin levels might be associated with the significant reduction of adipose tissue after IT surgery (Ramzy et al., 2014).

In addition, the beneficial effects of IT surgery on glucose metabolism have been considered to be the result of changes in gut hormones, such as GLP-1 and PYY, after surgery (Ramzy et al., 2014; Sun et al., 2014). Previous studies have demonstrated that circulating concentrations of GLP-1 and PYY in fasting or postprandial states were increased in GK rats or SD rats after IT surgery (Chen et al., 2011; Nausheen et al., 2013; Ramzy et al., 2014; Sun et al., 2014). However, in the present study, there were no differences in fasting serum levels of GLP-1 and PYY after IT surgery in GK rats. This inconsistency remains to be further investigated.

Interestingly, the Sham-IT surgery in the present study was first found to decrease body weight gain and food intake, reduce sWAT mass, improve glucose tolerance, and increase insulin sensitivity, as well as increase the expression of FGFR1, its co-receptor KLB and UCP1 in the sWAT of GK rats, suggesting that surgery *per se* might also have some beneficial effects on glucose and lipid metabolism. Sham surgery is also a surgical intervention that might induce the stress responses of organisms. Significant effects of sham laparotomy surgery or partial hepatectomy in Wistar rats were also found by other researchers, who considered that the effect of sham surgery might be a consequence of surgical stress or trauma (Werner et al., 2014).

In conclusion, IT surgery significantly decreased the body weight, fat mass and fasting blood glucose levels, as well as increasing the insulin sensitivity, of diabetic GK rats. These beneficial roles of IT surgery were probably associated with its stimulatory action on the expression of FGFR1 and its co-receptor KLB in both eWAT and pWAT and then promoting UCP1 expression in these tissues. Our findings could provide preliminary evidence to propose IT surgery as a novel and potential therapeutic method for diabetes. Further studies in a larger number of animals must be performed to explore the detailed mechanism.

## Author contributions

KY: Performed the molecular biology experiments, analyzed the data, and wrote the primary manuscript; WC: Performed the animal experiments; FG: Designed the experiment, supervised all of the experiments, and revised the primary manuscript; HZ, GL, and HP: Designed and supervised the experiments; NL, LW, and HY: Helped to perform the animal experiments and supervised the biochemical parameters measurements; ML: Analyzed the data.

### Conflict of interest statement

The authors declare that the research was conducted in the absence of any commercial or financial relationships that could be construed as a potential conflict of interest.

## Abbreviations

IT: ileal transposition
WAT: white adipose tissue
OGTT: oral glucose tolerance test
IPITT: intraperitoneal insulin tolerance test
FGF21: fibroblast growth factor 21
GLP-1: glucagon-like peptide 1
PYY: peptide YY
PPARα: peroxisome-proliferator-activated receptor-α
FGFR1: fibroblast growth factor receptor 1
KLB: β klotho
GYS2: glycogen synthase 2
UCP1: uncoupling protein 1
PGC1α: peroxisome proliferator-activated receptor gamma coactivator 1α
PRDM16: PR domain containing 16
CIDEA: cell death-inducing DNA fragmentation factor, alpha subunit-like effector A
Tmem26: transmembrane protein 26
Cox8b: cytochrome C oxidase subunit VIIIb
TNFα: tumor necrosis factor α
IL-6: interleukin-6.
