# Supplementary material for: Ileal Transposition Surgery Decreases Fat Mass and Improves Glucose Metabolism in Diabetic GK Rats: Possible Involvement of FGF21
Source: Front Physiol. 2018 Mar 9;9:191. doi: 10.3389/fphys.2018.00191 (PMC5854974; doi:10.3389/fphys.2018.00191)
Supplement: Supplementary file 1 [file Table1.DOCX]

**Supplementary Material**

**Table S1: Primers for RT-qPCR**

| FGFR1 | F: 5’-TGCTTGGCGGGTAACTCTATCG-3’ |
| --- | --- |
|  | R: 5’-CATCACGGCTGGTCTCTCTTCC-3’ |
| KLB | F: 5’-TTGTGGCTTGGTGAGTGG-3’ |
|  | R: 5’-CTGGGCATAGTGGTGTCC-3’ |
| FGF21 | F: 5’-CTCCTGCTGCCTGTCTTC-3’ |
|  | R: 5’-GTGTCCTGGTCGTCATCTG-3’ |
| UCP1 | F: 5’-CTCGGCTGGCTTGATGAC-3’ |
|  | R: 5’-TTCTGTGGTGGCTATAACTCTG-3’ |
| PGC1α | F: 5’-AGAGGCAGAAGCAGAAAGC-3’ |
|  | R: 5’-GTCACAGGTGTAACGGTAGG-3’ |
| PRDM16 | F: 5’-CGAGGAGACACGACTTGAG-3’ |
|  | R: 5’-GACCTATTCTACAGCATTGACC-3’ |
| CIDEA | F: 5’-CCTCGGCTGTCTCAATGTC-3’ |
|  | R: 5’-GGATGGCTGCTCTTCTGTG-3’ |
| Tmem26 | F: 5’-CGTAAGTGAAGAATGTGACAAC-3’ |
|  | R: 5’-AAACCCGTGGAGTAGATGG-3’ |
| Cox8b | F: 5’-GTGATTCCTAAAGCCCGTATC-3’ |
|  | R: 5’-ATGAAGCCAGCGATTATGAC-3’ |
| GYS2 | F: 5’-CCTCCTCAGTACCACCTTCC-3’ |
|  | R: 5’-TCAGCCTCCTCTTCCTCATC-3’ |
| PPARα | F: 5’- GGTCATACTCGCAGGAAAG-3’ |
|  | R: 5’-GCAGCAGTGGAAGAATCG-3’ |
| TNFα | F: 5’-TGTTCATCCGTTCTCTACC-3’ |
|  | R: 5’-CCACTACTTCAGCGTCTC-3’ |
| IL-6 | F: 5’-CCAACTTCCAATGCTCTCC-3’ |
|  | R: 5’-TCCACAAACTGATATGCTTAGG-3’ |
| β actin | F: 5’-ATCGGCAATGAGCGGTTC-3’ |
|  | R: 5’-ACTGTGTTGGCATAGAGGTC-3’ |

F: forward primer, R: reverse primer
