# Supplementary material for: Ileal Transposition Surgery Decreases Fat Mass and Improves Glucose Metabolism in Diabetic GK Rats: Possible Involvement of FGF21
Source: Front Physiol. 2018 Mar 9;9:191. doi: 10.3389/fphys.2018.00191 (PMC5854974; doi:10.3389/fphys.2018.00191)
Supplement: Supplementary file 2 [file Table2.DOCX]

**Supplementary Material**

**Table S2: Raw data of OGTT and IPITT**

|  | **No surgery (n=7)** | **Sham-IT (n=6)** | **IT (n=5)** |
| --- | --- | --- | --- |
| Glucose during OGTT (mmol/L) | | | |
| **0 min** | 13.96±0.55 | 11.95±0.24 ^*^ | 8.30±0.28 ^#^ |
| **30 min** | 20.39±0.42 | 16.65±0.46 ^*^ | 14.12±0.31 ^#^ |
| **60 min** | 17.87±0.43 | 15.54±0.46 ^*^ | 10.98±0.43 ^#^ |
| **120 min** | 14.77±0.58 | 14.05±0.44 | 8.6±0.20 ^#^ |
| Glucose during IPITT (mmol/L) | | | |
| **0 min** | 14.04±0.64 | 12.03±0.24 ^*^ | 8.42±0.24 ^#^ |
| **30 min** | 10.91±0.49 | 9.57±0.15 ^*^ | 5.18±0.35 ^#^ |
| **60 min** | 12.03±0.50 | 10.10±0.22 ^*^ | 5.66±0.45 ^#^ |
| **120 min** | 12.50±0.52 | 10.63±0.18 ^*^ | 6.32±0.33 ^#^ |

Values are the mean ± SEM. ^*^ *P*<0.05 *vs.* the No surgery group, ^#^ *P*<0.05 *vs*. the Sham-IT group.
